# Efficient production of multi-modified pigs for xenotransplantation by ‘combineering’, gene stacking and gene editing

**DOI:** 10.1038/srep29081

**Published:** 2016-06-29

**Authors:** Konrad Fischer, Simone Kraner-Scheiber, Björn Petersen, Beate Rieblinger, Anna Buermann, Tatiana Flisikowska, Krzysztof Flisikowski, Susanne Christan, Marlene Edlinger, Wiebke Baars, Mayuko Kurome, Valeri Zakhartchenko, Barbara Kessler, Elena Plotzki, Izabela Szczerbal, Marek Switonski, Joachim Denner, Eckhard Wolf, Reinhard Schwinzer, Heiner Niemann, Alexander Kind, Angelika Schnieke

**Affiliations:** 1 Chair of Livestock Biotechnology, School of Life Sciences Weihenstephan, Technische Universität München, Freising, Germany; 2 Institute of Farm Animal Genetics, FLI, Mariensee, Neustadt. Rbge, Germany; 3 Medizinische Hochschule Hannover, Transplantations labor, Hannover, Germany; 4 Chair of Molecular Animal Breeding and Biotechnology, Ludwig-Maximilians-Universität München, Oberschleissheim, Germany; 5 Robert Koch Institute, Berlin, Germany; 6 Department of Genetics and Animal breeding, Poznan University of Life Sciences, Poznan, Poland

## Abstract

Xenotransplantation from pigs could alleviate the shortage of human tissues and organs for transplantation. Means have been identified to overcome hyperacute rejection and acute vascular rejection mechanisms mounted by the recipient. The challenge is to combine multiple genetic modifications to enable normal animal breeding and meet the demand for transplants. We used two methods to colocate xenoprotective transgenes at one locus, sequential targeted transgene placement - ‘gene stacking’, and cointegration of multiple engineered large vectors - ‘combineering’, to generate pigs carrying modifications considered necessary to inhibit short to mid-term xenograft rejection. Pigs were generated by serial nuclear transfer and analysed at intermediate stages. Human complement inhibitors CD46, CD55 and CD59 were abundantly expressed in all tissues examined, human HO1 and human A20 were widely expressed. ZFN or CRISPR/Cas9 mediated homozygous *GGTA1* and *CMAH* knockout abolished α-Gal and Neu5Gc epitopes. Cells from multi-transgenic piglets showed complete protection against human complement-mediated lysis, even before *GGTA1* knockout. Blockade of endothelial activation reduced TNFα-induced E-selectin expression, IFNγ-induced MHC class-II upregulation and TNFα/cycloheximide caspase induction. Microbial analysis found no PERV-C, PCMV or 13 other infectious agents. These animals are a major advance towards clinical porcine xenotransplantation and demonstrate that livestock engineering has come of age.

Xenotransplantation from porcine donors could solve the severe shortage of several human tissues and organs available for transplantation, but pigs require numerous modifications to protect xenografts against the powerful rejection mechanisms mounted by the recipient.

Hyperacute rejection is initiated by pre-formed antibodies against endothelial α1,3-galactosyl-galactose (αGal) epitopes, resulting in complement activation and rapid graft destruction^1^,^2^. It can be overcome by genetic inactivation of the *GGTA1* (alpha-galactosyltransferase 1) gene^3^,^4^,^5^, or over-expression of human complement regulatory genes such as CD46, CD55 and CD59^6^,^7^,^8^. Protection is further improved by a combination of both^9^,^10^,^11^. Many transgenic pig lines carrying complement regulators have been generated but most contain one or two complement regulators, typically cDNAs or minigenes that often express poorly. There has been one report of pigs carrying three complement regulators, this was generated by microinjection of CD46 and CD59 constructs into a CD55 transgenic background, but transgene expression was neither ubiquitous, nor abundant^12^. Integration of transgenes at different genomic loci is also undesirable because segregation reduces the proportion of multi-transgenic offspring.

Acute vascular rejection (AVR) occurs within a few days and is characterised by procoagulant changes in the porcine endothelium and activation of complement and coagulation systems resulting in apoptosis, thrombosis, oedema and platelet aggregation in the graft^13^. The underlying mechanisms are incompletely understood, but antibodies to antigens other than αGal play an initiating role^14^,^15^. Complement regulators or *GGTA1* knockout do not inhibit AVR. The target for most human non-Gal xenoantibodies is the sialic acid N-glycolylneuraminic acid (Neu5Gc)^16^ synthesised by the *CMAH* (cytidine monophospho-N-acetylneuraminic acid hydroxylase) gene, which is inactive in humans. Porcine *CMAH* inactivation is thus required for clinical porcine xenotransplantation. The anti-apoptotic and anti-inflammatory genes A20 (tumour necrosis factor alpha-induced protein 3) and HO1 (heme oxygenase 1) also inhibit endothelial activation and AVR^17^,^18^.

Efficient genetic modification of farm animals became possible when somatic cell nuclear transfer enabled cell-mediated transgene addition and gene targeting, circumventing the lack of functional pluripotent stem cells^19^,^20^. The pace is now accelerating with continued improvements in nuclear transfer, synthetic endonucleases^21^ and improved genomic sequence data^22^ finally making important, life-saving applications such as xenotransplantation a reality.

We used various strategies to generate pigs carrying xenoprotective modifications designed to inhibit short- to mid-term porcine xenograft rejection. Sequential targeted gene placement - ‘gene stacking’ was investigated as a means of cointegrating transgenes and used to generate one line. Co-integration of multiple engineered high capacity vectors - ‘combineering’, with gene editing and serial nuclear transfer^5^,^23^ were used to generate the other lines described here. We report multi-transgenic pigs carrying genomic versions of human complement regulators CD46, CD55, CD59 plus cDNA cassettes for human A20 and HO1 to provide endothelium protection, with all transgenes at a single locus. Biallelic knockout of *GGTA1* and *CMAH* genes^24^ was then carried out in this multi-transgenic background. Evaluation of relevant viruses and microorganisms revealed the founder genotype to be free of the identified risk factors. This new generation of xenodonor animals should significantly increase the success of preclinical studies and advance progress to clinical xenotransplantation.

This work demonstrates that complex combined genetic modifications can now be efficiently engineered in livestock, and similar approaches taken in other areas such as large animal models of human diseases.

## Results

**Figure 1:**
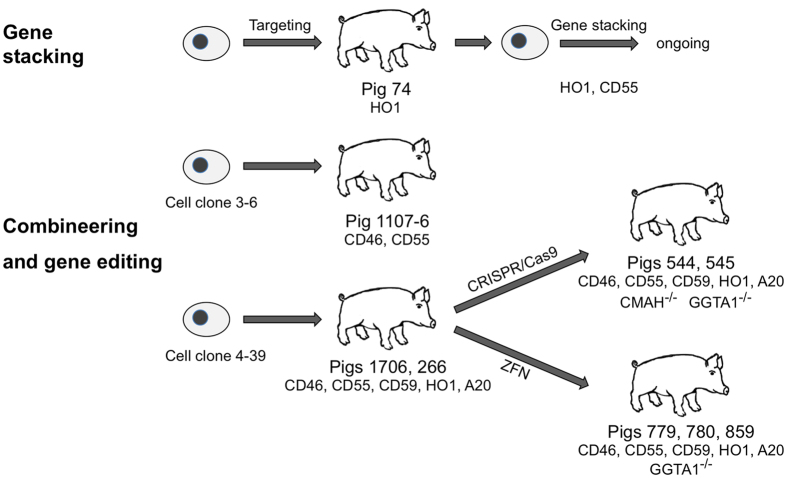


We wished to generate cells that support production of animals that ubiquitously express multiple xenoprotective transgenes from a single genomic locus. Two alternative strategies were adopted to assemble an array of colocated transgenes: sequential targeted transgene placement ‘gene stacking’ at a known permissive locus, and cointegration of engineered high capacity bacterial artificial chromosome (BAC) and phage artificial chromosome (PAC) vectors, ‘combineering’. Cell clones with characterised expression were then used to inactivate xenoreactive endogenous genes. Genetic manipulation schemes are summarised in Fig. 1.

### Transgene stacking at the porcine *ROSA26* locus

As in mice, the porcine *ROSA*26 locus provides a ‘safe harbour’ for transgene expression without interrupting the function of essential endogenous genes^25^. We previously demonstrated that porcine *ROSA26* could be targeted efficiently and support abundant ubiquitous transgene expression^26^. Here we placed an SV40-driven human HO1 cDNA into porcine *ROSA26* essentially as described for a reporter gene^26^ (Suppl. Fig. 1A). Approximately 5% of mesenchymal stem cell (MSC) cell clones were correctly targeted. Groups of clones were used for nuclear transfer, 177 reconstructed embryos were transferred to two recipient sows, one pregnancy established and one liveborn piglet (pig 74) obtained. This was sacrificed and HO1 detected in all tissues examined (Suppl. Fig. 1B). Porcine kidney fibroblasts (PKF) were derived and used for retargeting.

We generated a CAG promoter-driven CD55 minigene that showed expression levels similar to the average obtained with genomic CD55 in MSCs. A second targeting vector was used to place the human CD55 minigene 5′ to the HO1 cassette and exchange the selectable marker (Suppl. Fig. 1A). Targeted cell clones were again derived with high efficiency (11% of clones analysed) and groups of clones used for nuclear transfer. Nuclear transfer is ongoing, one pregnancy has been established and piglets expected soon.

### One step generation of multi-transgenic pigs

We also investigated co-placement of multiple transgenes at random loci because this allowed the use of large genomic constructs. Co-transfection of several DNA constructs commonly results in integration at a single genomic locus. Combining multiple transgenes within the same vector further increases the probability of co-localisation and co-expression. While a permissive integration site could not be predetermined, cell clones could be screened for expression before nuclear transfer, because complement regulators are naturally expressed in MSCs. Serial nuclear transfer was used to check expression *in vivo* and then regenerate viable animals. Figure 1 outlines the schemes used.

Rounds of construct development were first carried out to maximise transgene expression in porcine MSCs (summarised in Methods section). MSC cell clones were then derived by co-transfection of either two or three constructs: a) human genomic CD46 BAC and human genomic CD55 BAC; b) human genomic CD46 BAC, human genomic CD59 PAC, and a triple-transgene BAC with CAG-driven human genomic CD55, SV40-driven human HO1 cDNA and CAG-driven human A20 cDNA (Suppl. Fig. 2). Several hundred transfected MSC cell clones were screened for transgene expression by RT-PCR, and quantified relative to human MSC line SCP1, which expresses all complement regulators^27^. CD46 mRNA expression was highest in clone 3–6 (>7 fold higher than SCP1). Clone 4–39 expressed all five transgenes, with especially high levels of CD55 (>90 fold higher than SCP1) considerably greater than any other clone analysed. These were used for nuclear transfer.

For cell clone 3–6, 253 reconstructed embryos were transferred to three recipients, one pregnancy was established and one stillborn piglet obtained: CD46, CD55 double transgenic animal ID 1107-6. For cell clone 4–39, 119 reconstructed embryos were transferred to one recipient, a pregnancy established and one live-born piglet obtained: CD46, CD55, CD59 A20, HO1 multi-transgenic animal ID 1706. Piglet 1706 was healthy and developed normally, but died due to injury. Organ samples were collected for expression analysis and PKFs cultured for functional analysis, further rounds of genetic manipulation, and nuclear transfer to regenerate each animal.

For re-cloning of piglet 1107-6, 348 reconstructed embryos were transferred to four recipients, two pregnancies were established, and 11 liveborn and one stillborn offspring obtained. One piglet died two weeks *post partum*. For re-cloning of piglet 1706, 212 reconstructed embryos were transferred to two recipients, one pregnancy established and one liveborn offspring (pig 266) obtained. All surviving animals remain healthy, and we now have F1 generation animals from both lines.

### PERV and microbe screening

Xenografts should pose minimal infectious risk to human recipients. We carried out genome and serum analysis of multi-transgenic pig 266 (recloned from pig 1706), which formed the basis for subsequent genetic modifications and found it to be free of PERV-C expression. This animal was also free of other microbial infections. Details are shown in Suppl. Fig. 8.

### Multi-transgenes are inserted at a single locus

The integration sites of the two constructs in 1107-6 cells, and the three constructs in 1706 cells were determined by FISH analysis. In each case transgenes were co-located at single genomic loci. The transgene locus in 1706 has been identified as chromosome 6q22. (Suppl. Fig. 3; Suppl. Fig. 4A,B).

### Expression and xenoprotective function of transgenes and genetic knockouts

**Figure 2:**
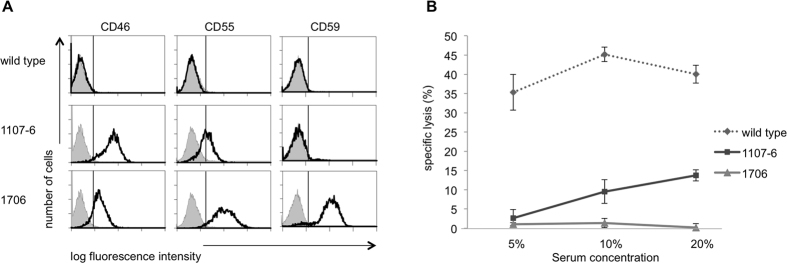
(**A**) Transgene expression in porcine kidney fibroblasts. Flow cytometry analysis of human transgenes CD46, CD55, and CD59 (solid line) in kidney derived fibroblasts (PKF). (**1**) wild-type; (**2**) piglet 1107-6; (**3**) piglet 1706. Grey histograms indicate secondary antibody staining only. (**B)** Protection of multi-transgenic porcine fibroblasts from complement-mediated lysis. 51-Cr labelled PKFs from wild-type, double transgenic 1107-6 (CD46, CD55), and multi-transgenic 1706 (CD46, CD55, CD59, A20, HO1) animals incubated with concentrations of human serum as indicated. Shown is % specific lysis (mean ± SD) calculated from triplicate samples. Data are representative of four independent experiments.

Lung, liver, spleen, kidney, heart and aorta were analysed for hCD46, hCD55 and hCD59 mRNA expression. Representative RT-PCR results for multi-transgenic animal 1706 are shown in Suppl. Fig. 5A, and Q-RT-PCR of various organs in Suppl. Fig. 5B. Consistent with data from the original MSC cell clones, hCD46 expression was highest in double transgenic animal 1107-6; multi-transgenic animal 1706 showed high levels of hCD55 followed by hCD59 and somewhat less hCD46. Relative protein quantification by FACS analysis of PKFs confirmed these findings (Fig. 2A).

Protection against human complement-mediated lysis was tested by incubation with concentrations of human serum from 2.5% to 20% (Fig. 2B). Double transgenic 1107-6 PKFs showed markedly less lysis than wild-type, and multi-transgenic 1706 PKFs were completely protected at the highest serum concentration, even though they were still αGal positive.

### Derivation of multi-transgenic and *GGTA1* knockout animals

PKFs from piglet 1706 were modified by inactivating *GGTA1* using a zinc-finger nuclease targeted to exon 8, as previously described^28^. *GGTA1*-deficient cells were counter-selected with isolectin B4 (IB4), which specifically binds αGal, and the resultant population confirmed as αGal free by flow cytometry. These multi-transgenic, *GGTA1*-deficient cells were again used for nuclear transfer. 563 reconstructed embryos were transferred to six recipients and three pregnancies established, two of which continued to term. Three liveborn piglets (779, 780, 859) and one stillborn piglet were obtained. Pig 779 was euthanised after three months due to polyarthritis. Pigs 780 and 859 developed normally, continue to thrive and are being used to found a pig line.

*GGTA1 s*equence analysis of pigs 779, 780 and 859 revealed that all carried a 1 bp and a 5 bp deletion within the ZFN target region in each allele.

### Multi-transgenic *GGTA1* knockout pigs express xenoprotective genes including their isoforms and are resistant to complement-induced lysis

**Figure 3:**
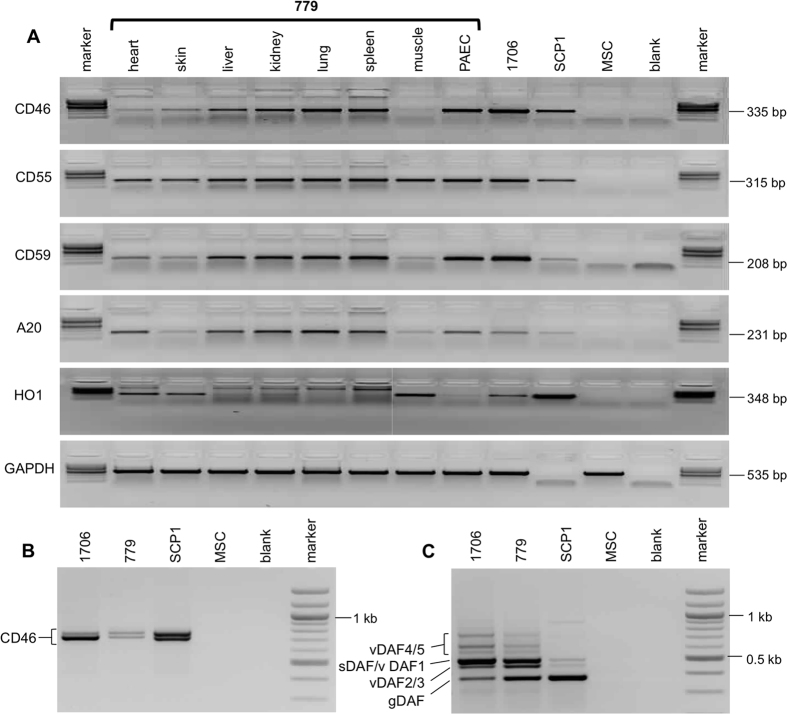
(**A**) RT-PCR analysis of multi-transgenic *GGTA1*-deficient piglet 779 organs and cultured endothelial cells. Organs from piglet 779, piglet 1706 PKF, human MSC line SCP1 and wild-type porcine MSCs are indicated. Please note that the GAPDH primers used were specific to porcine samples, so human SCP1 showed no amplification. (**B**) RT-PCR analysis of CD46 splicing variants. PKFs from multi-transgenic piglet 1706 and multi-transgenic *GGTA1*-deficient piglet 779, human MSC line SCP1 and wild-type porcine MSCs are as indicated (**C**) RT-PCR analysis of CD55 RNA splicing variants. Lanes are as in B. RT-PCR bands indicated correspond to membrane-bound CD55 isoforms: gDAF, vDAF4 and vDAF5 and the soluble isoforms sDAF, vDAF1, vDAF2 and vDAF3 in PKF of transgenic pigs 1706 and 779 as described in normal human tissues^26^.

Analysis of animal 779 revealed hCD46, hCD55, hCD59 and hA20 mRNA expression in all organs analysed (Fig. 3A). Human HO1 expression was evident in heart, skin and muscle, with lower levels in liver, kidney, spleen and aortic endothelial cells (PAEC), but not detected in lung.

Human CD46 and CD55 are normally expressed as several RNA splicing variants encoding membrane-bound and soluble protein isoforms that vary by tissue^29^, and thought to be required for full biological activity^30^,^31^. Figure 3B and C show RT-PCR detection of RNA splicing variants in 1706 PKFs and *GGTA1*-deficient 779 PKFs. Human CD46 amplified a ~760 bp fragment consistent with expression of splice variants hCD46-002, 004, 005 and 006 (names according to Ensembl database) and a 805 bp fragment consistent with hCD46-001 and hCD46-007. Samples amplified from pig 1706 were isolated, subcloned and the DNA sequence determined, generating sequences consistent with hCD46 splice variants 002/004 and 005/006 from the 760 bp fragment and sequences consistent with hCD46 splice variants 001/007 from the 805 bp fragment (Suppl. Seq. file 1). HCD55 amplified fragments closely similar to splice variants observed in normal human tissue, encoding membrane-bound protein isoforms gDAF, vDAF4 and vDAF5 and soluble isoforms sDAF, vDAF1, vDAF2 and vDAF3^29^. Fragments representing the two major splice variant RNAs encoding membrane-bound (gDAF, hCD55-001) and soluble (sDAF, hCD55-009) CD55 isoforms were subcloned and the sequences determined confirming their identity, (Suppl. Seq. file 2). Most hCD59 splice variants encode the same 128 amino acid protein isoform (Ensembl), so it was not tested. Western analysis of 1706 PKFs revealed expression of hCD46 56 kDa and 66 kDa isoforms, the hCD55 70 kDa isoform and 43 kDa, 46 kDa precursors, and the hCD59 25 kDa protein (Suppl. Fig. 6).

**Figure 4:**
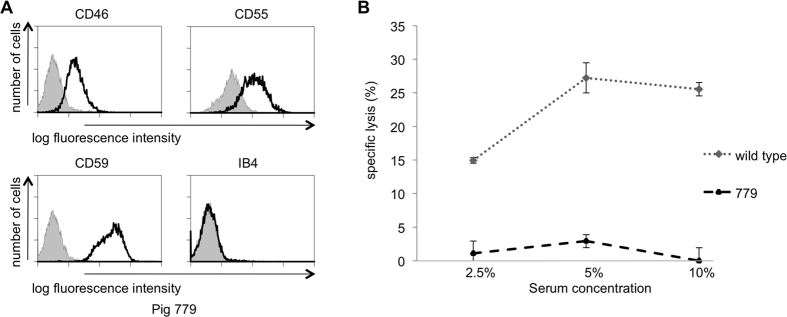
Phenotypic and functional analysis of multi-transgenic porcine *GGTA1*-deficient fibroblasts. (**A**) Flow cytometry analysis of human CD46, CD55 and CD59 expression and loss of α-Gal epitopes in piglet 779 PKFs. Grey histograms represent secondary antibody staining only. (**B**) 51-Cr labelled PKF from wild-type and multi-transgenic *GGTA1*-deficient piglet 779 were incubated with concentrations of human serum as indicated. Shown is % specific lysis (mean ± SD) calculated from triplicate samples of a representative experiment.

FACS analysis of blood from multi-transgenic *GGTA1*-deficient piglets 779 and 780 revealed complement regulator expression and the absence of αGal antigens, consistent with homozygous *GGTA1* inactivation (Fig. 4A). Piglet 779 PDKFs showed similar resistance to human complement-mediated lysis as multi-transgenic *GGTA1*-intact 1706 cells, indicating that abundant complement regulator expression already provides substantial protection (Figs 2B and 4B).

### A20 and HO1 provide vascular protection

**Figure 5:**
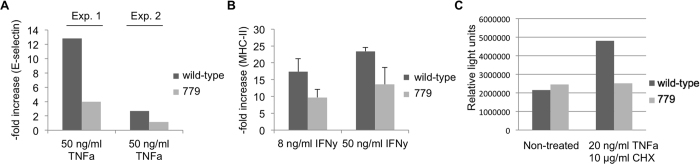
Reduced cytokine-induced upregulation of E-selectin and MHC class-II and caspase 8 induction in multi-transgenic *GGTA1* KO porcine aortic endothelial cells. Porcine aortic endothelial cells from a wild-type animal and multi-transgenic *GGTA1*-deficient piglet 779 were cultured with medium or cytokines. (**A**) Upregulation of E-selectin after 2 days exposure to 50 ng/mL TNFα. (**B**) Upregulation of porcine MHC class-II after 3 days stimulation with 8 ng/mL or 50 ng/mL IFNγ (N = 3). p < 0.05, two-tailed unpaired t-test. Data are shown as mean ± standard deviation (SD). (**C**) Luminescence assay for induction of caspase 8 activity in kidney fibroblasts after 24 h 20 ng/mL TNFα 10 μg/mL cycloheximide.

We examined the functional effects of human A20 and HO1 expression. A20 and HO1 both have anti-apopotic effects via inhibition of caspase activity. After treatment with 20 ng/mL human TNFα and 10 μg/mL cycloheximide, PKFs from multi-transgenic pig 1706 showed a 3% increase of caspase activity while that in wild-type PKFs increased by 124% (Fig. 5C).

We also investigated effects on cytokine-induced upregulation of adhesion molecules. Human A20 also inhibits NFκB-mediated expression of E-selectin. TNFα-induced upregulation of E-selectin in PAECs from multi-transgenic *GGTA1*-deficient pig 779 was diminished by 58–69% compared to wild-type cells (Fig. 5A). The ability of human HO1 to inhibit IFNγ mediated upregulation of porcine MHC class-II molecules was tested in 779 PAECs by treatment with 8 ng/ml and 50 ng/ml IFNγ. PAECs of pig 779 showed 42–44% (p < 0.05) lower induction of MHC class-II expression than wild-type cells (Fig. 5B). We cannot exclude that A20 expression did not contribute to the observed results.

### *CMAH/GGTA1* double knockout

**Figure 6:**
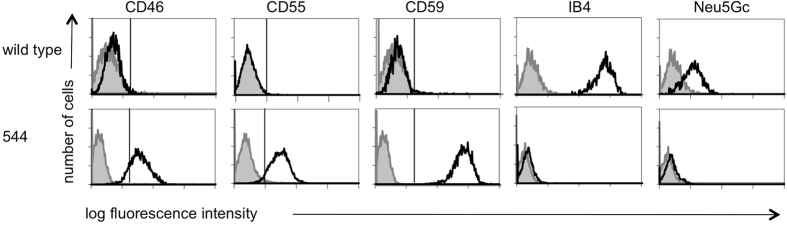
Phenotypic analysis of multi-transgenic porcine *GGTA1, CMAH* double deficient fibroblasts. Flow cytometry analysis of human CD46, CD55, CD59 expression and loss of α-Gal and Neu5Gc epitopes in piglet 544 PKFs. Grey histograms represent secondary antibody staining only.

To generate *CMAH/GGTA1* double knockout animals in the 1706 multi-transgenic background we used CRISPR/Cas9 gene editing with serial nuclear transfer. Homozygous *CMAH*-knockout 1706 PKF cell clones were identified by sequence analysis across the target site. A clone that carried a single base insertion 3bp 5′ of the PAM motif and an 11bp deletion in the other allele was used for nuclear transfer and two pregnancies established, one was terminated at 27 days, three foetuses explanted and PKFs derived. These were subjected to *GGTA1* knockout by CRISPR/Cas9. A cell clone carrying an 11bp deletion in one allele and an 18 bp insertion in the other was identified and used for nuclear transfer. 403 reconstructed embryos were transferred to four recipients, two pregnancies were established, and two liveborn offspring (544 and 545) obtained. One piglet (544) died six days after birth. PKFs were isolated and used for FACS analysis and western blotting, confirming *CMAH/GGTA1* double knockout (Fig. 6 and Suppl. Fig. 7). To facilitate breeding of the multi-transgenic *CMAH*/*GGTA1* double knockout animals once they are available, we have also created female *CMAH*/*GGTA1* double knockout animals using a female PKF clone carrying a homozygous C insertion in *GGTA1* exon 8, and a double heterozygous T insertion, 14 bp deletion in *CMAH* exon 10. Two healthy normal females (488, 490; Suppl. Fig. 7) are currently being raised for mating.

## Discussion

Inhibition of hyperacute and acute-vascular rejection are required for long-term porcine xenograft survival in primates. Here we report pigs with a series of genetic modifications designed to address both.

Gene stacking has so far only been performed using site-specific recombinases^32^. We show that it is also possible to add two transgenes (CD55 and HO1) as single copies by successive rounds of homologous recombination at the porcine *ROSA26* locus, and obtain high ubiquitous expression of each. Further transgenes can easily be added to the array, and it will be interesting to find out how many can be stacked while maintaining the permissive nature of porcine *ROSA26*.

The use of *E. coli* based homologous recombination systems, developed for functional genomics in mice^33^, was key to our engineering of large BAC and PAC constructs incorporating multiple xenoprotective transgenes. To ensure independent expression of each transgene we used separate expression constructs. Alternative means of combining smaller components e.g. cDNAs, such as 2A peptide or IRES-based polycistronic systems, have also been used to produce multiple transgenic xenodonor pigs^34^,^35^, but these carry a risk of poor or variable expression^36^.

Serial nuclear transfer was key to the strategy we employed as it enabled sequential transgene addition and gene editing steps to generate pigs carrying the battery of modifications necessary to provide robust xenograft protection.

We adopted a multipronged approach to blocking hyperacute rejection. Three important regulators, CD46, CD55 and CD59 were used to inhibit complement activation at the levels of C3 convertase, C5 convertase, and the membrane attack complex. Such combined inhibition prevents cross-activation of the complement system at stages later than C3 convertase, for example by coagulation factors that interact with the complement system^30^. The transgenes used were genomic sequences, rather than cDNAs or minigenes, to facilitate abundant expression and enable expression of CD46 and CD55 RNA splice variants and the protein isoforms necessary for full biological function^31^,^37^. Challenge with human complement revealed that a combination of CD46 and CD55 markedly reduced lysis, and that CD46, CD55 and CD59 together provided essentially complete protection *in vitro*. These results compare very favourably with a previous report of triple complement regulator transgenic pigs that described 20% lysis of PBMCs after one hour incubation^12^, compared to complete protection after four hours in our assay.

Our *in vitro* lysis assay discerned no additional benefit from *GGTA1* gene inactivation in the multi-transgenic background, but the situation *in vivo* will likely be more rigorous. Pig-to-primate xenotransplantation showed that protection against hyperacute rejection is increased when *GGTA1* inactivation is combined with complement regulator expression^38^. *GGTA1*-KO xenografts are however still subject to acute vascular rejection, characterised by endothelial activation probably by preformed non-Gal antibodies, causing thrombotic microangiopathy^39^. This led us to include the anti-inflammatory and anti-apoptotic genes hHO1 and hA20 as vascular protectors. We also inactivated the *CMAH* gene responsible for the major non-Gal antigen Neu5Gc (Hanganutziu-Deicher)^40^.

Heme oxygenase-1 has a range of anti-apoptotic and anti-inflammatory effects. It reduces NK cell activity and formation of pro-inflammatory factors such as CCR5 (C-C chemokine receptor type 5), ICAM-1 (intercellular adhesion molecule 1), VCAM-1 (vascular cell adhesion molecule 1) and E-selectin (CD62 antigen-like family member E)^41^,^42^. It inhibits platelet aggregation, maintaining microcirculation and facilitating angiogenesis^43^. In rodent xenografts HO1 reduces thrombus formation and IgM deposition^44^ and prolongs organ survival^45^,^46^. In pigs HO1 protects endothelial cells from TNF-α-mediated apoptosis, extends *ex vivo* perfusion of kidneys with human blood^18^, protects fibroblasts from H_2_O_2_ damage, and inhibits TNF-α and cycloheximide-mediated apoptosis^47^. Our analysis revealed wide but not ubiquitous HO1 expression, and found that cytokine-induced MHC-class II up-regulation in aortic endothelial cells was reduced by almost one half. This is very encouraging, and could be due to co-expression of hHO1 with hA20.

A20 inhibits NF-κB activation^48^ and thus upregulation of pro-inflammatory and pro-apoptotic cytokines including TNFα and interleukin-1β (IL-1β)^49^. A20 protects porcine aortic endothelial cells against TNFα-induced apoptosis and confers partial protection against ischemia/reperfusion injury^17^. We found ubiquitous A20 expression, and E-selectin upregulation an indicator of NF-κB activation was markedly reduced (65% <wild-type) in aortic endothelial cells. Again, this could be due to co-expression of hA20 with hHO1. The anti-apoptotic effects of A20 and HO1 were also confirmed by inhibition of caspase 8 induction.

Safe, effective xenotransplantation requires that donor animals do not transmit zoonotic pathogens. We examined the founder multi-transgenic pig 226 and found it to be to be free of a series of known and potential infectious agents, including expressed PERV-C. Inactivation of all PERV loci in a porcine cell line *in vitro* has been reported recently^50^, but whether such multiple gene editing is compatible with the production of viable pigs has yet to be established.

We are now breeding multi-modified *GGTA1*-deficient boars 780 and 859 with homozygous *GGTA1*-deficient sows to enable *ex vivo* perfusion of explanted organs, and transplantation into primates. *CMAH/GGTA1* double knockout sows 488 and 490 are also available for breeding with multi-transgenic double knockout males.

There have been several recent successes in pig-to-primate xenotransplants using multi-transgenic porcine organs, with record survival times of 125 and 136 days for kidneys^51^,^52^ and a remarkable 945 days for hearts^53^. These donors were all *GGTA1*-deficient, and expressed one or two complement and one coagulation regulator in the organ used. The combination of genetic modifications we describe is the most extensive reported so far, and should extend xenograft survival further, but might not yet be complete. The full requirements for clinically effective xenotransplantation will become evident as transplant studies continue and salient aspects of delayed xenograft rejection are revealed. Although CD46 affects coagulation and fibrinolytic cascades^54^ further transgenes may be required to combat coagulation dysregulation and thrombotic microangiopathy associated with endothelial activation. Candidate transgenes for future inclusion thus include human anticoagulant thrombomodulin^55^, the endothelium protein C receptor^56^ and CD39^57^. The adaptive immune response will present the next challenge, and human recipients will probably require immunosuppression. There is encouraging and unanticipated evidence that *GGTA1* inactivation and CD46 expression down-regulate the human T-cell response to pig cells *in vitro*^58^,^59^, nevertheless T-cell regulators such as CTLA4-Ig/LEA29Y^60^ may be necessary. It is eminently feasible to add additional transgenes adjacent to the existing array. Maintaining a single locus is important to simplify breeding of donor animals to meet the demand for xenotransplants.

## Methods

Animal experiments were approved by the Government of Upper Bavaria (permit number 55.2-1-54-2532-6-13) or by the Lower Saxony State Office for Consumer Protection and Food Safety (LAVES permit number 33.14-42502-04-12/0891) and performed according to the German Animal Welfare Act and European Union Normative for Care and Use of Experimental Animals.

### Reagents

Chemicals were obtained from Sigma-Aldrich Chemie GmbH unless otherwise specified, cell culture media and supplements were obtained from Thermo Fisher Scientific or Invitrogen unless otherwise specified, antibodies were purchased from Santa Cruz unless otherwise specified.

### Transgene stacking at the porcine *ROSA26* locus

The ROSA26 HO1 targeting vector consisted of: a 2.2 kb short homology arm; splice acceptor, promoterless neo; SV40 driven HO1 cDNA^18^; and a 4.7 kb long homology arm (Suppl. Fig. 1A). Targeting in MSCs was carried out by standard methods and confirmed in cell clones by long-range 5′ and 3′ junction PCR and sequence analysis (PCR primers shown in Suppl. Table 1). The ROSA26 CD55 retargeting vector consisted of the same 2.2 kb short homology arm; splice acceptor; promoterless blasticidin resistance; a CAG-driven CD55 minigene composed of CD55 exon 1, intron 1 and exon2 ligated at the HindIII site within exon 2 to CD55 cDNA (membrane bound form); and a 5 kb long homology arm (Suppl. Fig. 1A). Targeting was carried out in PKF from a ROSA26 HO1 targeted piglet by standard methods, confirmed in cell clones by long range 5′ and 3′ junction PCR and sequence analysis (PCR primers shown in Suppl. Table 1).

### Modification of complement regulatory gene constructs

Bacterial artificial chromosome (BAC) vectors containing genomic sequences of human CD46 and CD55, and a phage artificial chromosome (PAC) vector containing human genomic CD59 were purchased from Source BioScience, UK (CD46 BAC: RP11-99A19; CD55 BAC: RP11-357P18; CD59 PAC: RP4-541C22). Superfluous regions were deleted and drug-selectable cassettes PGK/Em7-driven neomycin resistance (Neo) or SV40/EM7-driven blasticidin resistance (BS) inserted by homologous recombination in *E. coli* strain SW106 (recombineering) by standard methods^61^. Each molecular clone was then reduced in size by successive restriction digestion and recombineering stages and tested for expression in porcine MSCs to identify a suitable configuration to be introduced into pigs.

The large CD46 BAC construct, which consisted of a 66 kb 5′ flanking/promoter region, 43 kb CD46 gene and 54 kb 3′ flanking region was reduced by deleting distal regions of the 3′ flank to generate ‘medium’ and ‘small’ constructs with 34 kb and 7 kb 3′ flanks. The large hCD46 construct provided best expression and was chosen to generate pigs.

The large CD59 PAC construct consisting of 10 kb 5′ flanking/promoter region, 34 kb CD59 gene and 82 kb 3′ flanking region was reduced at the distal 3′ flanking region to ‘medium’ and ‘small’ constructs with 57 kb and 37 kb 3′ flanks. The ‘small’ version provided abundant expression and so was used to generate pigs.

The large CD55 BAC construct consisting of 28 kb 5′ flanking/promoter sequence, 40 kb CD55 gene and 103 kb 3′ flanking region was reduced at the distal 3′ flanking region to ‘medium’ and ‘small’ constructs with 33 kb and 6 kb 3′ flanks. The 5′ flanking/promoter region of the ‘small’ construct was then reduced to 10 kb to generate an ‘extra small’ CD55 construct. A further construct, termed CAG-CD55, was generated in which the 5′ flanking/promoter region was replaced by a 1.8 kb CAG synthetic promoter (CMV enhancer, chicken beta-actin promoter, rabbit beta-globin splice acceptor). The ‘extra small’ CD55 and CAG-CD55 constructs were chosen on the basis of abundant expression *in vitro*. CAG-driven hA20 cDNA^17^ and SV40-driven hHO1 cDNA cassettes^18^ were then combined into a single vector and added to the CAG-CD55 BAC at a location 3′ of the CD55 3′ flank by recombineering using two 500bp homologous arms derived from the CD55 3′ flank. Outline structures of the constructs used to generate pigs are shown in Suppl. Fig. 2.

### Cell isolation, culture and transfection

Mesenchymal stem cells (MSC) were isolated from adipose tissue from German Landrace pigs and cultured as described^62^. Samples of 1 × 10^7^ MSCs were co-transfected using Lipofectamine 2000 (Life Tech., USA) with 10–30 μg DNA composed of either a mixture of CD46 and CD55 (endogenous promoter) constructs in 1:1 molar ratio, or a mixture of hCD46, hCD59 and hCD55-hA20-hHO1 constructs in 1:1:1 molar ratio, cell clones were selected and isolated by standard methods.

Porcine kidney fibroblasts (PKF) were isolated and cultured by standard methods. Porcine aortic endothelial cells (PAEC) were isolated as described^17^ and cultured in DMEM, 20% FCS, 4 mM glutamine, 200 U/mL penicillin, 200 μg/mL streptomycin, 25 mM HEPES, 50 μg/mL endothelial cell growth supplement (Corning, USA) at 37 °C and 10% CO_2_, adjusted to 5% CO_2_ during cytokine stimulation. Porcine peripheral blood mononuclear cells (pPBMC) were isolated from heparinised blood samples by Ficoll density gradient centrifugation.

### Somatic cell nuclear transfer

Nuclear transfer was performed as described^63^,^64^. In short, donor cells were arrested at G0/G1 of the cell cycle by serum deprivation, a single cell was inserted into the perivitelline space of enucleated *in vitro* matured oocytes from prepubertal gilts, then fused and oocytes activated by electric pulse. Reconstructed embryos were transferred into oviducts of hormonally synchronised recipient gilts by mid-ventral laparotomy.

### RNA isolation and detection of expression

RNA was isolated and cDNA synthesised by standard methods. RT-PCR primer sequences are shown in Suppl. Table 1. QRT-PCR was carried out using the TaqMan Fast Universal PCR Master Mix (Life Tech., USA) with 5′FAM/3′BHQ labelled probes (MWG Eurofins Operon, Germany). Each sample was measured three times using MicroAmp Fast Optical 96-Well Reaction Plates (Life Tech., USA) and a 7500 Fast Real-Time PCR Cycler.

### Western analysis

Protein was isolated and Western analysis carried out by standard methods. Human CD55 was detected using rabbit anti-CD55 HPA 02190 (diluted 1:200) and horseradish peroxidase (HRP) labelled anti-rabbit A9161 (diluted 1:5000). Human CD46 was detected using rabbit anti-CD46 sc-9098 (diluted 1:200) and HRP labelled anti-rabbit A9161 (diluted 1:5000). Human CD59 was detected using goat anti-CD59 (C-19) sc-7076 (diluted 1:500) and donkey anti-goat IgG-HRP sc-2020, (diluted 1:5000), Neu5Gc epitopes were detected using purified anti-Neu5Gc antibody (clone Poly21469 chicken IgY, BioLegend, USA; diluted 1:10000 in Neu5GC free blocking solution) and HRP labelled goat anti-chicken sc-2428 (diluted 1:5000 in Neu5GC free blocking solution). GAPDH was detected using mouse monoclonal anti-GAPDH #G8795, (diluted 1:3000) and rabbit anti-mouse IgG H&L (HRP) ab6728 (diluted 1:5000).

### Fluorescence *in situ* hybridisation (FISH)

Prior to hybridization, chromosomes were Q-banded using 0.005% quinacrine mustard solution for 90 s. A standard FISH protocol was applied^65^, using three genomic probes: hCD59 labelled with DEAC-5-dUTP (PerkinElmer, USA), hCD55 with dig-11-dUTP (Roche, Germany), and hCD46 with biotin-16-dUTP (Roche, Germany). In each case mean probe fragment size was ~500 bp. Digoxigenin-labelled probes were detected with anti-digoxigenin-fluorescein Fab fragments (Roche). Chromosome preparations were mounted in Vectashield (Vector Laboratories) with DAPI counterstain and analysed under a Nikon Ni-U fluorescence microscope. International nomenclature for identification of pig chromosomes was applied^66^.

### *GGTA1* and *CMAH* gene inactivation

Plasmids encoding a zinc-finger nuclease (ZFN) targeted to *GGTA1* exon 8 were electroporated by standard methods. αGal-negative cells were enriched by counter-selection with streptavidin-coated magnetic beads and biotin-conjugated isolectin B4 (Dynabeads, Life Technologies, USA) in a magnetic field^28^. *GGTA1* gene editing was analysed by allele-specific sequencing of cloned PCR fragments amplified across the target site.

*GGTA1*/*CMAH* double knockout was performed in two steps. A CRISPR/Cas9 enzyme targeted to the sequence 5′ GGAAGAAACTCCTGAACTACA 3′ in *CMAH* exon 10 was transfected into 1706 cells, and cell clones analysed by allele-specific sequencing of cloned PCR fragments amplified across the target site (Suppl. Table 1). Homozygous knockout clones were used for nuclear transfer, a pregnancy was terminated at day 28, foetal fibroblasts isolated and transfected with a CRISPR/Cas9 enzyme targeted to the sequence 5′ GACGAGTTCACCTACGAG 3′ in *GGTA1* exon 8. αGal-negative cells were counter-selected and verified by sequencing as above.

### Immunofluorescence staining and flow cytometry

0.1–0.5 × 10^6^ cells were incubated with one of the following antibodies: anti-human CD46-PE (TRA-2-10, BioLegend, USA), anti-human CD55-bio (IA10, BD Biosciences, USA), anti-human CD59-PE (p282, BioLegend, USA), anti-porcine CD62e FITC (1.2B6, Acris, USA), anti-porcine MHC class-II (MSA-3, kindly provided by Prof. Dr. Saalmüller, Austria). Binding of biotinylated primary antibodies was visualised with streptavidin-PE (BD Biosciences, USA), and binding of unlabelled primary reagents visualised with goat anti-mouse IgG-FITC secondary antibody (Dianova, Germany). PAEC quality was evaluated with anti-rat CD31 mAb, which cross-reacts with porcine CD31 (TLD-3A12, BD Biosciences, USA). The α1,3-Gal epitope was detected with tetrameric isolectin B4-FITC (Enzo Life Sciences, Germany) and the Neu5Gc epitope detected using purified anti-Neu5Gc antibody (clone Poly21469, BioLegend, USA) and FITC conjugated donkey anti-chicken IgY (Jackson ImmunoResearch, USA). Cells were analysed on a FACSCalibur flow cytometer (BD Biosciences, USA) with CellQuest Pro Software (BD Biosciences, USA). Dead cells were excluded from the analysis.

### Caspase assay

Kidney fibroblasts were incubated for 24 h with 20 ng/mL human TNFα (Biomol GmbH, Germany) and 10 μg/mL cycloheximide (Sigma-Aldrich, Germany). Cells were detached and caspase 8 activity measured using the Promega Caspase-Glo 8 assay according to the manufacturer’s protocol.

### Assay for complement-mediated lysis of porcine fibroblasts

51-Chromium release assays were performed as described^25^. 1 × 10^4^ cells/well were incubated with 2.5%, 5%, 10% or 20% pooled complement-preserved normal human serum (Dunn Labortechnik, Germany). After 4 hours, 25 μL cell supernatant were removed and radioactivity measured in a Microbeta scintillation counter (Wallac, Finland). Mean cpm of triplicate cultures was used for all calculations. Spontaneous ^51^Cr release was determined by incubation with medium alone, and maximum release by incubation with 2% Triton X-100. Specific lysis was calculated as: % specific lysis = (experimental ^51^Cr release – spontaneous ^51^Cr release)/(maximum ^51^Cr release – spontaneous ^51^Cr release) × 100.

### Cytokine-induced upregulation of E-selectin and MHC class-II

PAECs (passages 2–5) were stimulated for 2 days with 50 ng/mL human TNFα (Biomol GmbH, Germany) then analysed for E-selectin (CD62e) expression, or stimulated for 3 days with 8 ng/mL or 50 ng/mL porcine IFNγ (R+D systems) then analysed for porcine MHC class-II upregulation by flow cytometry. CD62e or MHC class-II upregulation was calculated as fold-increase mean fluorescence intensity normalised to background staining and relative to untreated cells. Statistical analysis was performed with GraphPad Prism 6 (Graph-Pad Software, USA) and significance was calculated using two-tailed unpaired t-test.

### Screening for PERV, HEV, CMV and other microorganisms

Please see details accompanying Suppl. Fig. 8.

## Additional Information

**How to cite this article**: Fischer, K. *et al.* Efficient production of multi-modified pigs for xenotransplantation by ‘combineering’, gene stacking and gene editing. *Sci. Rep.* **6**, 29081; doi: 10.1038/srep29081 (2016).

## Supplementary Material

Supplementary Information
