# Supplementary material for: Efficient production of multi-modified pigs for xenotransplantation by ‘combineering’, gene stacking and gene editing
Source: Sci Rep. 2016 Jun 29;6:29081. doi: 10.1038/srep29081 (PMC4926246; doi:10.1038/srep29081)
Supplement: Supplementary Information [file srep29081-s1.pdf]

Supplementary Information

Efficient production of multi-modified pigs for xenotransplantation by 'combineering', gene stacking and gene editing.

Konrad Fischer, Simone Kraner-Scheiber, Björn Petersen, Beate Rieblinger, Anna Buermann, Tatiana Flisikowska, Krzysztof Flisikowski, Susanne Christan, Marlene Edlinger, Wiebke Baars, Mayuko Kurome, Valeri Zakhartchenko, Barbara Kessler, Elena Plotzki, Izabela Szczerbal, Marek Switonski, Joachim Denner, Eckhard Wolf, Reinhard Schwinzer, Heiner Niemann, Alexander Kind, Angelika Schnieke

Supplementary Figures 1 - 8
Supplementary Tables 1 and 2
Supplementary Sequence Files 1 and 2

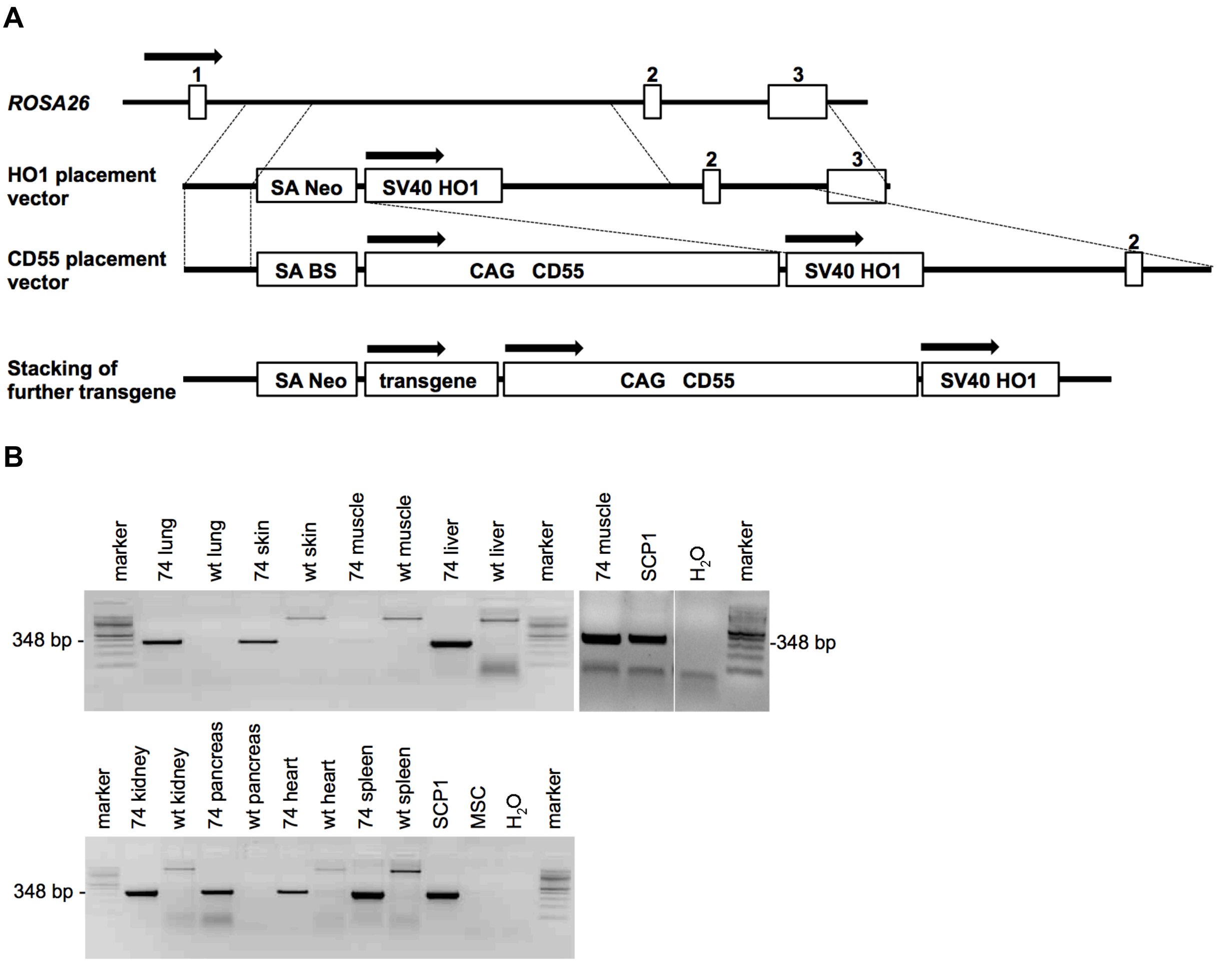

Supplementary Figure 1

Gene stacking.

A. Top, Porcine *ROSA26* locus, exons are indicated by boxes and numbered as indicated. Upper middle, Placement of SV40 driven HO1 cassette into *ROSA26*. Lower middle, Retargeting to place a CAG driven CD55 minigene adjacent to HO1. Bottom, Sequential retargeting to stack further transgenes and exchange the drug resistance marker. Dotted lines indicate regions of homology in targeting vectors. SA splice acceptor. Transcription is indicated by arrows.

B. RT-PCR analysis of HO1 expression in organs of pig 74, which carries the SV40-driven HO1 cDNA placed at the ROSA26 locus, compared to wild-type pig organs as shown. MSC, primary porcine mesenchymal stem cells; human MSC line SCP1 used as a positive control; water PCR control, as indicated. The size of the 348 bp diagnostic fragment is marked. Note: The 74 muscle lane was underloaded in the left panel, an extra panel on the right confirms HO1 expression in muscle.

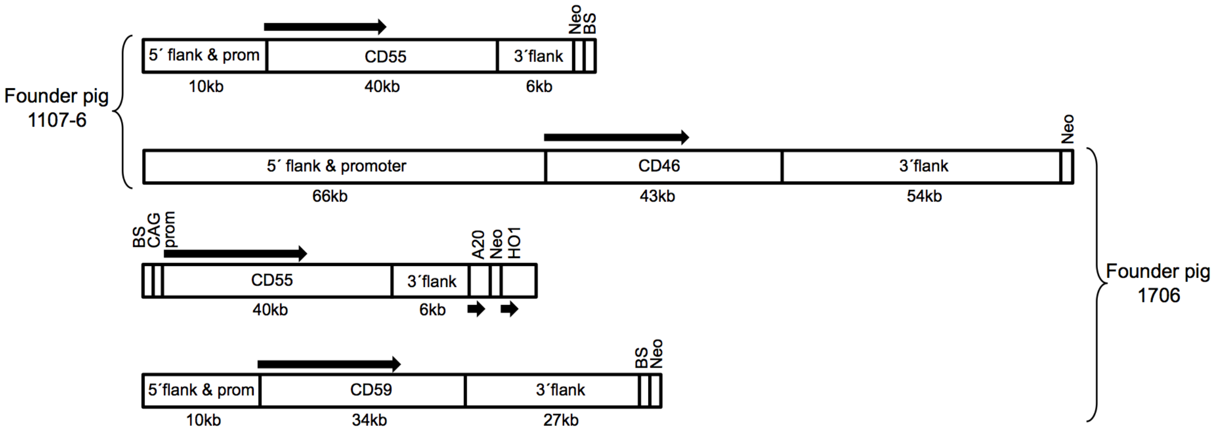

Supplementary Figure 2

Outline structure of constructs containing genomic complement regulator sequences and endothelial protection cDNA expression cassettes. Transcription is indicated by arrows and the position of neomycin and blasticidin selection cassettes shown. The combination of constructs in each of the founders generated by 'combineering' is indicated by brackets.

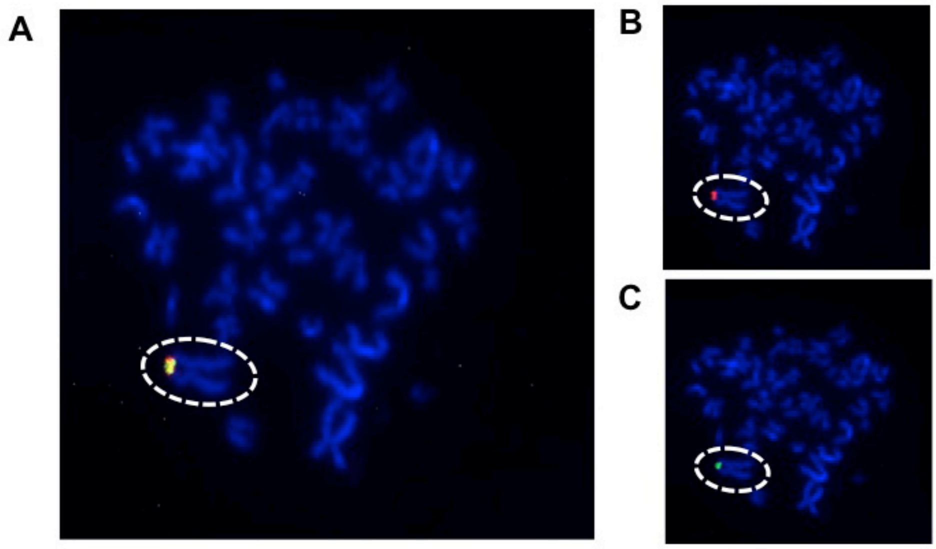

Supplementary Figure 3

A, Fluorescent in situ hybridisation analysis of piglet 1107-6. Overlay of CD46 and CD55 signals. **B,** CD46 (Cy3.5) signal. **C,** CD55 (fluorescein) signal.

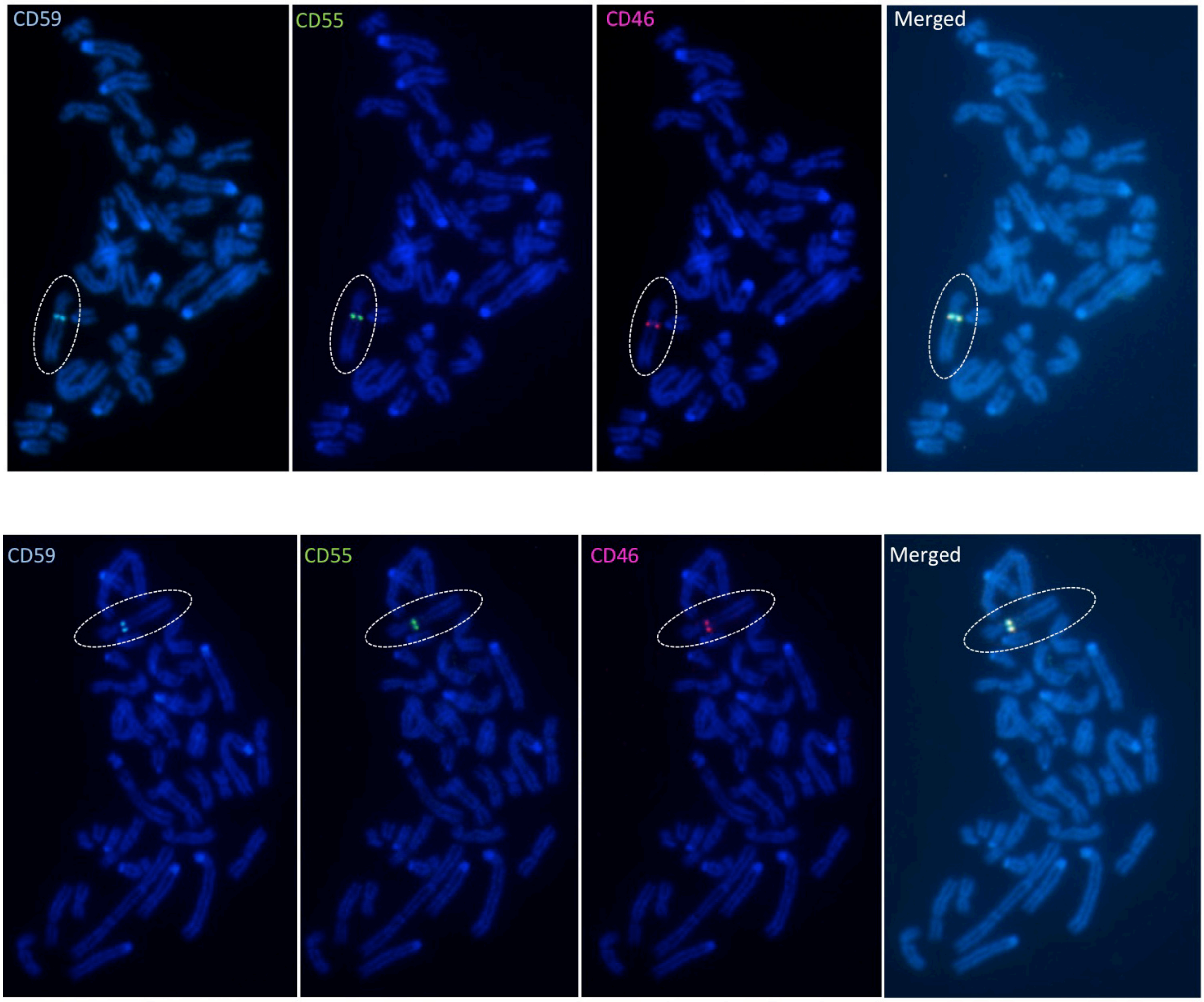

Supplementary Figure 4 A

Fluorescent in situ hybridisation analysis of multi-transgenic piglet 1706.

Sample metaphase spreads with CD46 (Cy3.5), CD55 (fluorescein), CD59 (DEAC) signals and merged overlay, as indicated.

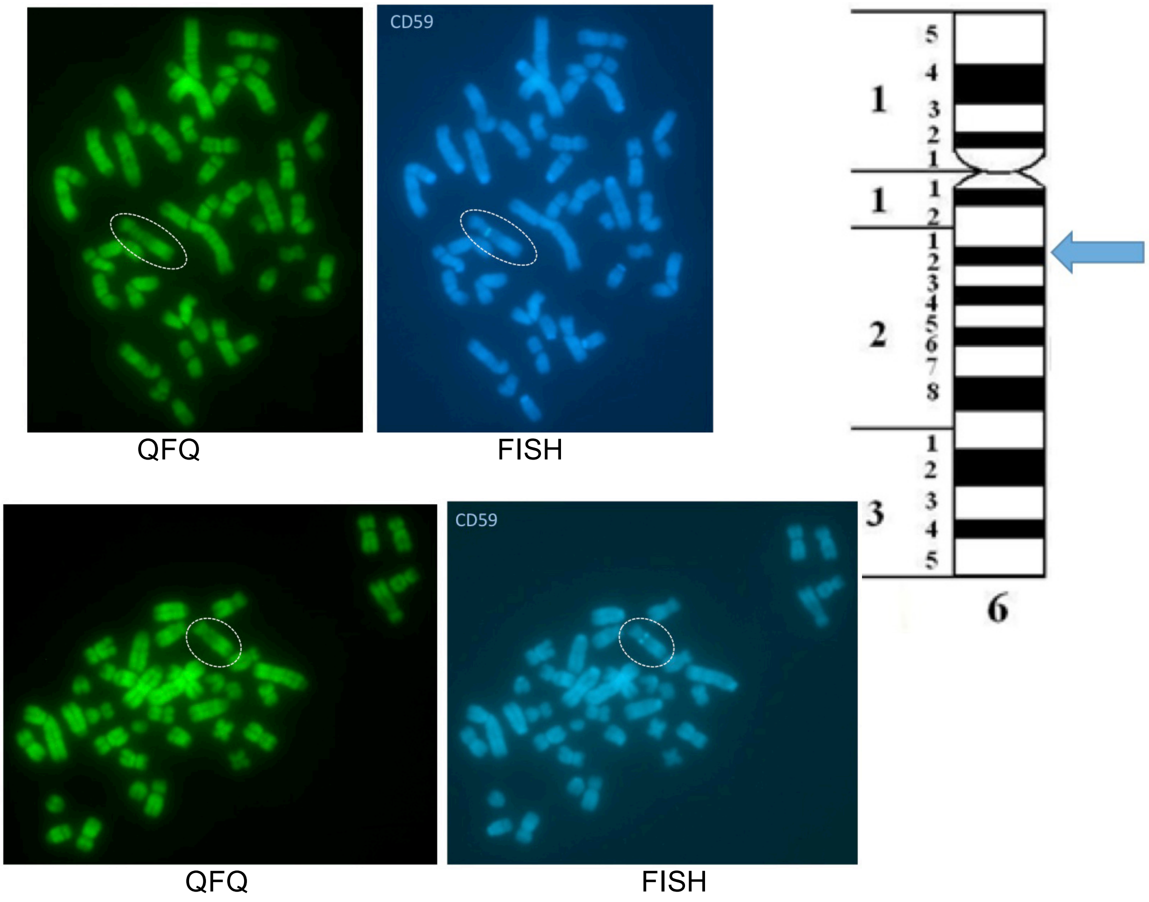

Supplementary Figure 4 B

Q banding and fluorescent in situ hybridisation analysis of multi-transgenic piglet 1706.

Sample metaphase spreads showing localisation of CD59 on porcine chromosome 6 q2 2.

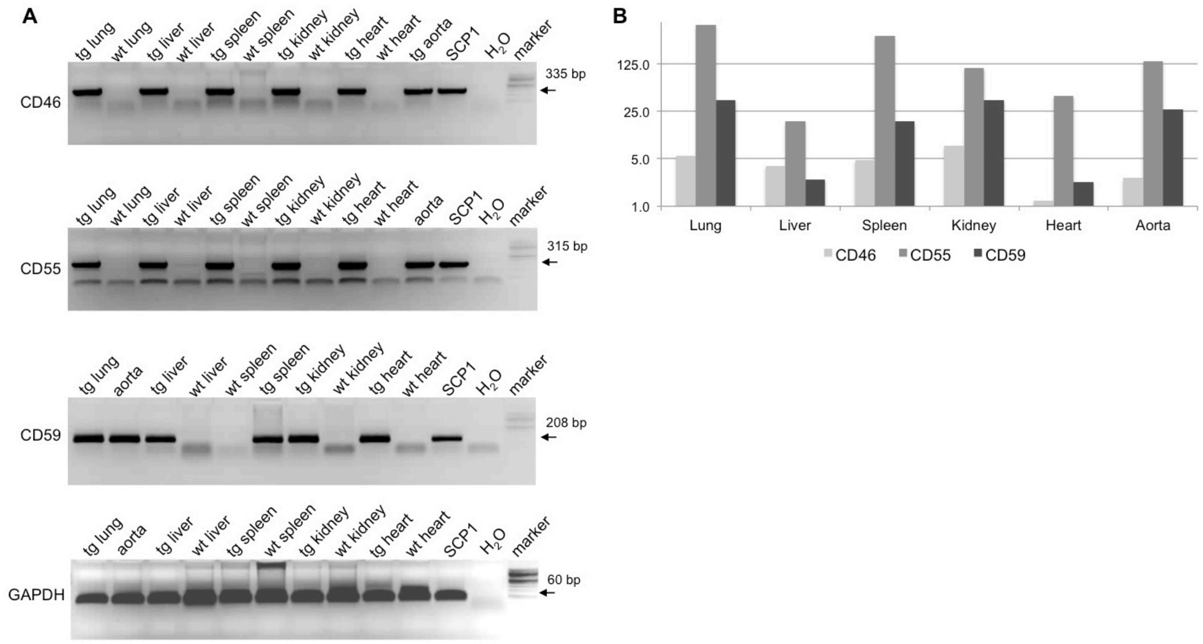

Supplementary Figure 5

A, RT-PCR analysis of CD46, CD55 and CD59 expression in multi-transgenic piglet 1706 organs. 1706 multi-transgenic (tg) and wild-type (wt) lung, aorta, liver, spleen, kidney and heart as indicated. Human MSC line SCP1 was used as a positive control.

B, Q-RT-PCR analysis of CD46, CD55 and CD59 expression in organs of multi-transgenic piglet 1706. Expression values are shown relative to those in human MSC line SCP1 (expression=1). Please note that the y-axis indicates 5-fold differences of expression.

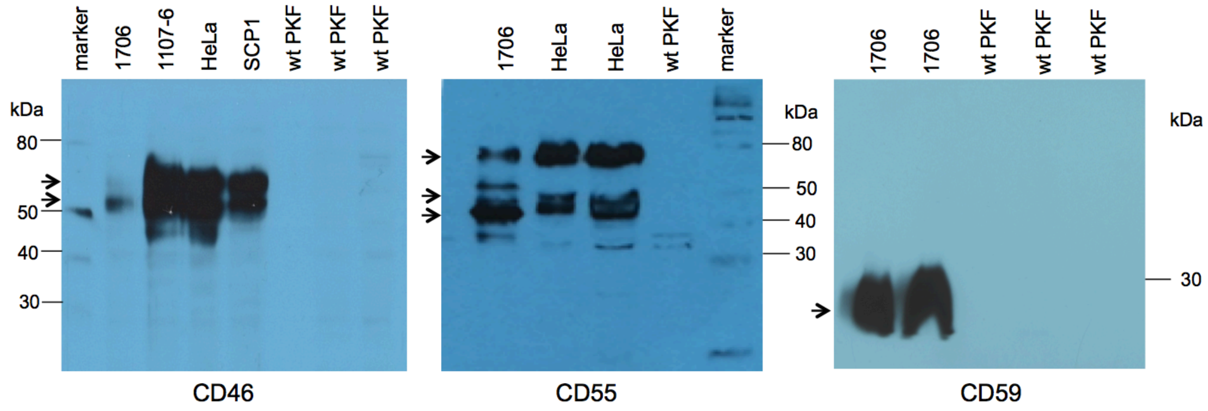

Supplementary Figure 6

Western blot analysis of complement regulator expression in PKF of multi-transgenic piglet 1706. Detection of CD46, CD55 and CD59 in cell types as indicated.

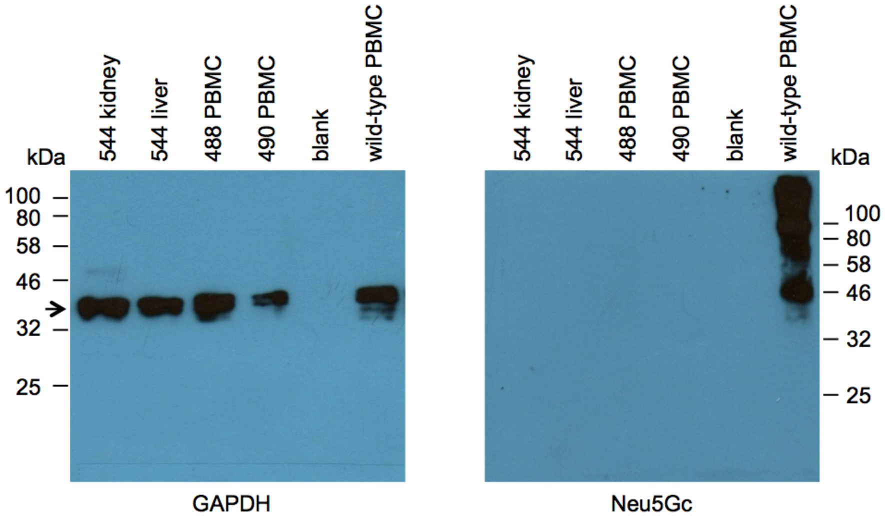

Supplementary Figure 7

Western blot analysis of CMAH deficient pigs. Shown are organs from *CMAH/GGTA1* double knockout multi-transgenic male piglet 544 and blood cells from *CMAH/GGTA1* double knockout non-transgenic females 488 and 490. PBMC peripheral blood mononuclear cells. Note that wild-type PBMC show a range of proteins carrying N-glycolylneuraminic acid epitopes.

Supplementary Figure 8

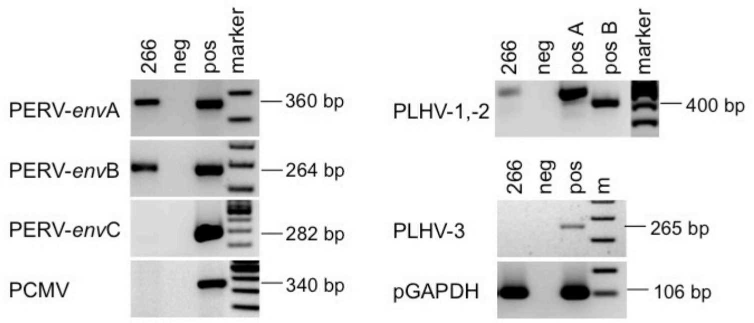

PCR analysis of porcine endogenous retroviruses (PERV), porcine cytomegalovirus (PCMV) and porcine lymphotropic herpesvirus (PLHV) in multi-transgenic cells of pig 266 (recloned pig 1706). Human 239 cells were used as negative control. Positive control A for PLHV-1, positive control B for PLHV-2, M, marker, the size of the amplicon is indicated.

Microbiological analysis of founder pig 266

The possibility of activating porcine endogenous retroviruses (PERV) has long been recognized, although evidence is growing that the risk posed is probably negligible (1). Nevertheless, following the precautionary principle, we examined the founder multi-transgenic pig 226 for PERVs. PERV-A and PERV-B are present in all pigs, whereas the PERV-C load varies by breed (2) and is of concern because it can recombine with PERV-A to generate infectious recombinant PERV-A/C (3). We found that the genome of founder 266 was free of PERV-C (Suppl. Fig. 8).

Risks posed by other agents are clearer. PCMV, a herpes virus, reduces survival of pig-to-baboon and pig-to-cynomolgus kidney transplants (3-5). Real time PCR analysis revealed that it was absent in the founder 266 genome.

Real-time PCR detected HEV sequences in 266, but blood analysis revealed no antibodies that would indicate exposure to infectious particles. Any risk posed by HEV genotype 3, the version present in pigs, is also unclear but means of eliminating it have been developed (1).

Porcine lymphotropic herpesvirus-1 (PLHV1) was detected in 266 serum, but PLHV2 and PLHV3 were not. PLHV1 is a γ-herpesvirus associated with post-transplant lymphoproliferative disorder in pigs (6), however the importance of these viruses for xenotransplantation is currently not known.

Also confirmed absent in 266 were: influenza virus, porcine parvovirus, porcine reproductive and respiratory syndrome virus, salmonella, porcine circovirus 2, actinobacillus pleuropneumoniae, mycoplasma hyopneumoniae (enzootic pneumonia), lawsonia intracellularis (porcine intestinal adenomatosus), chlamydia and leptospira.

Methods for Suppl Fig 8

DNA was isolated from EDTA blood using the DNAeasy blood and tissue kit (Qiagen, Germany), RNA was isolated from serum using the RNAeasy Minikit (Qiagen, Germany), according to the manufacturer´s instructions. PCR, real-time PCR and real-time RT-PCR for PERV-A, PERV-B, PERV-C, HEV, PCMV and PLHV1, 2, 3 and Western blot analysis for antibodies against HEV were performed as described (7) (Suppl. Table 2). Duplex real-time PCR to detect PCMV with primers specific for PCMV and porcine GAPDH as a reference was also carried out (Suppl. Table 2) using TaqMan Universal PCR 2x Mastermix (Life Technologies, USA) according to the manufacturer´s instructions. PCR to detect PERV-C were performed using the Kapa2G robust PCR Kit (Peqlab, Germany). Screening for other infectious agents was carried out by the Tiergesundheitsdienst Bayern e.V. (Bavarian Animal Health Service).

References - Suppl Fig 8

1. Denner, J. Xenotransplantation and hepatitis E virus. *Xenotransplantation* **22**, 167-173 (2015).

2. Denner, J. and Tönjes, R.R. Infection barriers to successful xenotransplantation focusing on porcine endogenous retroviruses. *Clin Microbiol Re*v. **25**, 318-343 (2012).

3. Denner, J. Xenotransplantation and porcine cytomegalovirus (PCMV). *Xenotransplantation* **22**, 329-335 (2015).

4. Yamada, K. *et al*. Porcine cytomegalovirus infection is associated with early rejection of kidney grafts in a pig to baboon xenotransplantation model. *Transplantation* **98**, 411-418 (2014).

5. Sekijima, M., *et al*. Results of life-supporting galactosyltransferase knockout kidneys in cynomolgus monkeys using two different sources of galactosyltransferase knockout swine. *Transplantatio*n **98**, 419-426 (2014).

6. Doucette, K., *et al*. Gene expression of porcine lymphotrophic herpesvirus-1 in miniature swine with post transplant lymphoproliferative disorder. *Transplantation* **83**, 87-90 (2007).

7. Plotzki, E., *et al*. Virus safety of islet cell transplantation from transgenic pigs to marmosets. *Virus Res.* **204**, 95-102 (2015).

Supplementary Table 1

Primer combinations for: RT-PCR analysis of transgene expression; screening of cell clones for *GGTA1* and *CMAH* gene inactivation; and junction PCR analysis of HO1 and CD55 transgene stacking at porcine *ROSA26* locus.

	Forward 5´-3´	Reverse 5´-3´	Size
CD46	CAAGCAGTCCCTGCAAATGG	GCAGCACGACTCCACACTGA	335 bp
CD55	TTGTCCAGCACCACCACAAA	CGTGTTGCTTGAGCATTTGG	315 bp
CD59	GGAGTTGAGACCTACTTCACAG	CTTTGGTAATGAGACACGCAT	208 bp
A20	TGGGACTCCAGAAAACAAGG	GTCCTTTTGGCCTCATGAAA	231 bp
HO1	GATGGAGCGTCCGCAACC	CTTCACATAGCGCTGCATGGC	348 bp
GAPDH	GGCGTGAACCATGAGAAGTATG	GGTGCAGGAGGCATTGCT	60 bp
GAPDH	TGGTGAAGGTCGGAGTGAAC	ATGAGGTCCACCACCCTGTT	535 bp
CD46 splice variants	CAAGCAGTCCCTGCAAATGG	TGCCTTTCTTCTTCCTCCTTTGA	
CD55 splice variants	GGATTCACCATGATTGGAGAGCACTC	AAGTCAGCAAGCCCATGGTTACTAGC	
GGTA1 screening	AAGACCATCGGGGAGCACAT	GGCTTTCATCATGCCACTCG	346 bp
CMAH screening	TGCCGTAAACAAAGAGGGATT	TTGTCTGCTGGGTGGGATTC	357 bp
ROSA26 HO1 5' junction	TATGGGCGGGATTCTTTTGC	GGAGCGGCGATACCGTAAAG	3.4 Kb
ROSA26 HO1 3' junction	GCCCCAGGATTTGTCAGAGG	CAGGTGGAAAGCTACCCTAGCC	6.9 Kb
ROSA26 CD55 5' junction	TATGGGCGGGATTCTTTTGC	CGGCTGTCCATCACTGTCCT	3.0 Kb
ROSA26 CD55 3' junction	TCCCACCAACAGTTCAGAAACCT	CAGGTGGAAAGCTACCCTAGCC	9.3 Kb

Supplementary Table 2

PCR and real-time PCR primers and probes used for microbiological analysis

	Forward 5´-3´	Reverse 5´-3´
PervenvA	TGGAAAGATTGGCAACAGCG	AGTGATGTTAGGCTCAGTGG
PervenvB	TTCTCCTTTGTCAATTCCGG	TACTTTATCGGGTCCCACTG
PervenvC	CTGACCTGGATTAGAACTGG	ATGTTAGAGGATGGTCCTGG
envC real	CCCCAACCCAAGGACCAG	AAGTTTTGCCCCCATTTTAGT
pCyclo real	TGCTTTCACAGAATAATTCCAGGATTTA	GACTTGCCACCAGTGCCATTA
pGAPDH	ACATGGCCTCCAAGGAGTAAGA	GATCGAGTTGGGGCTGTGACT
JVHEV	GGTGGTTTCTGGGGTGAC	TGATTCTCAGCCCTTCGC
PCMV real	GTTCTGGGATTCCGAGGTTG	ACTTCGTCGCAGCTCATCTGA
PCMV 199	ACGAGAAAGATATTCTGACGGTGCA	TCTAGACGAAAGGACATTGTTGATA
PLHV-1,-2 747	CAYGGTAGTATTTATTCAGACA	GATATCCTGGTACATTGGAAAG
PLHV-3 905	ACAAGAGCCTTAGGGTTCCAAACT	GTGTCCAGTGTTGTAATGGATGCC
envC probe	FAM-CTCTAACATAACTTCTGGATCAGACCC-BHQ1	
pCyclo probe	Cy5-TGCCAGGGTGGTGACTTCACACGCC-BHQ1	
pGAPDH probe	HEX-CCACCAACCCCAGCAAGAG-BHQ1	
JVHEVProbe	FAM-TGATTCTCAGCCCTTCGC-BHQ1	
PCMV real probe	FAM-CAGGGCGGCGGTCGAGCTC-TAMRA	

```
CD46-001/007   TATCAGATGCACTTTATTTGTAATGAGGGTTATTACTTAATTGGTGAAGAAATTCTATAT
seq            --------GCACTTTATTTGTAATGAGGGTTATTACTTAATTGGTGAAGAAATTCTATAT
                       ****************************************************

CD46-001/007   TGTGAACTTAAAGGATCAGTAGCAATTTGGAGCGGTAAGCCCCCAATATGTGAAAAGGTT
seq            TGTGAACTTAAAGGATCAGTAGCAATTTGGAGCGGTAAGCCCCCAATATGTGAAAAGGTT
               ************************************************************

CD46-001/007   TTGTGTACACCACCTCCAAAAATAAAAAATGGAAAACACACCTTTAGTGAAGTAGAAGTA
seq            TTGTGTACACCACCTCCAAAAATAAAAAATGGAAAACACACCTTTAGTGAAGTAGAAGTA
               ************************************************************

CD46-001/007   TTTGAGTATCTTGATGCAGTAACTTATAGTTGTGATCCTGCACCTGGACCAGATCCATTT
seq            TTTGAGTATCTTGATGCAGTAACTTATAGTTGTGATCCTGCACCTGGACCAGATCCATTT
               ************************************************************

CD46-001/007   TCACTTATTGGAGAGAGCACGATTTATTGTGGTGACAATTCAGTGTGGAGTCGTGCTGCT
seq            TCACTTATTGGAGAGAGCACGATTTATTGTGGTGACAATTCAGTGTGGAGTCGTGCTGCT
               ************************************************************

CD46-001/007   CCAGAGTGTAAAGTGGTCAAATGTCGATTTCCAGTAGTCGAAAATGGAAAACAGATATCA
seq            CCAGAGTGTAAAGTGGTCAAATGTCGATTTCCAGTAGTCGAAAATGGAAAACAGATATCA
               ************************************************************

CD46-001/007   GGATTTGGAAAAAAATTTTACTACAAAGCAACAGTTATGTTTGAATGCGATAAGGGTTTT
seq            GGATTTGGAAAAAAATTTTACTACAAAGCAACAGTTATGTTTGAATGCGATAAGGGTTTT
               ************************************************************

CD46-001/007   TACCTCGATGGCAGCGACACAATTGTCTGTGACAGTAACAGTACTTGGGATCCCCCAGTT
seq            TACCTCGATGGCAGCGACACAATTGTCTGTGACAGTAACAGTACTTGGGATCCCCCAGTT
               ************************************************************

CD46-001/007   CCAAAGTGTCTTAAAGTGCTGCCTCCATCTAGTACAAAACCTCCAGCTTTGAGTCATTCA
seq            CCAAAGTGTCTTAAAGTGCTGCCTCCATCTAGTACAAAACCTCCAGCTTTGAGTCATTCA
               ************************************************************

CD46-001/007   GTGTCGACTTCTTCCACTACAAAATCTCCAGCGTCCAGTGCCTCAGGTCCTAGGCCTACT
seq            GTGTCGACTTCTTCCACTACAAAATCTCCAGCGTCCAGTGCCTCAGGTCCTAGGCCTACT
               ************************************************************

CD46-001/007   TACAAGCCTCCAGTCTCAAATTATCCAGGATATCCTAAACCTGAGGAAGGAATACTTGAC
seq            TACAAGCCTCCAGTCTCAAATTATCCAGGATATCCTAAACCTGAGGAAGGAATACTTGAC
               ************************************************************

CD46-001/007   AGTTTGGATGTTTGGGTCATTGCTGTGATTGTTATTGCCATAGTTGTTGGAGTTGCAGTA
seq            AGTTTGGATGTTTGGGTCATTGCTGTGATTGTTATTGCCATAGTTGTTGGAGTTGCAGTA
               ************************************************************

CD46-001/007   ATTTGTGTTGTCCCGTACAGATATCTTCAAAGGAGGAAGAAGAAAGGCACATACCTAACT
seq            ATTTGTGTTGTCCCGTACAGATATCTTCAAAGGAGGAAGAAAAAAGG-------------
               *****************************************.*****
```

```
CD46-002/004   TATCAGATGCACTTTATTTGTAATGAGGGTTATTACTTAATTGGTGAAGAAATTCTATAT
seq            ------ATGCACTTTATTTGT-ATGAGGGTTATTACTTAATTGGTGAAGAAATTCTATAT
                     *************** **************************************

CD46-002/004   TGTGAACTTAAAGGATCAGTAGCAATTTGGAGCGGTAAGCCCCCAATATGTGAAAAGGTT
seq            TGTGAACTTAAAGGATCAGTAGCAATTTGGAGCGGTAAGCCCCCAATATGTGAAAAGGTT
               ************************************************************

CD46-002/004   TTGTGTACACCACCTCCAAAAATAAAAAATGGAAAACACACCTTTAGTGAAGTAGAAGTA
seq            TTGTGTACACCACCTCCAAAAATAAAAAATGGAAAACACACCTTTAGTGAAGTAGAAGTA
               ************************************************************

CD46-002/004   TTTGAGTATCTTGATGCAGTAACTTATAGTTGTGATCCTGCACCTGGACCAGATCCATTT
seq            TTTGAGTATCTTGATGCAGTAACTTATAGTTGTGATCCTGCACCTGGACCAGATCCATTT
               ************************************************************

CD46-002/004   TCACTTATTGGAGAGAGCACGATTTATTGTGGTGACAATTCAGTGTGGAGTCGTGCTGCT
seq            TCACTTATTGGAGAGAGCACGATTTATTGTGGTGACAATTCAGTGTGGAGTCGTGCTGCT
               ************************************************************

CD46-002/004   CCAGAGTGTAAAGTGGTCAAATGTCGATTTCCAGTAGTCGAAAATGGAAAACAGATATCA
seq            CCAGAGTGTAAAGTGGTCAAATGTCGATTTCCAGTAGTCGAAAATGGAAAACAGATATCA
               ************************************************************

CD46-002/004   GGATTTGGAAAAAAATTTTACTACAAAGCAACAGTTATGTTTGAATGCGATAAGGGTTTT
seq            GGATTTGGAAAAAAATTTTACTACAAAGCAACAGTTATGTTTGAATGCGATAAGGGTTTT
               ************************************************************

CD46-002/004   TACCTCGATGGCAGCGACACAATTGTCTGTGACAGTAACAGTACTTGGGATCCCCCAGTT
seq            TACCTCGATGGCAGCGACACAATTGTCTGTGACAGTAACAGTACTTGGGATCCCCCAGTT
               ************************************************************

CD46-002/004   CCAAAGTGTCTTAAAGTGTCGACTTCTTCCACTACAAAATCTCCAGCGTCCAGTGCCTCA
seq            CCAAAGTGTCTTAAAGTGTCGACTTCTTCCACTACAAAATCTCCAGCGTCCAGTGCCTCA
               ************************************************************

CD46-002/004   GGTCCTAGGCCTACTTACAAGCCTCCAGTCTCAAATTATCCAGGATATCCTAAACCTGAG
seq            GGTCCTAGGCCTACTTACAAGCCTCCAGTCTCAAATTATCCAGGATATCCTAAACCTGAG
               ************************************************************

CD46-002/004   GAAGGAATACTTGACAGTTTGGATGTTTGGGTCATTGCTGTGATTGTTATTGCCATAGTT
seq            GAAGGAATACTTGACAGTTTGGATGTTTGGGTCATTGCTGTGATTGTTATTGCCATAGTT
               ************************************************************

CD46-002/004   GTTGGAGTTGCAGTAATTTGTGTTGTCCCGTACAGATATCTTCAAAGGAGGAAGAAGAAA
seq            GTTGGAGTTGCAGTAATTTGTGTTGTCCCGTACAGATATCTTCAAAGGAGGAAGAAGAAA
               ************************************************************

CD46-002/004   GGGAAAGCAGATGGTGGAGCTGAATATGCCACTTACCAGACTAAATCAACCACTCCAGCA
seq            GGCAAA------------------------------------------------------
               ** ***
```

```
CD46-005/006   TATCAGATGCACTTTATTTGTAATGAGGGTTATTACTTAATTGGTGAAGAAATTCTATAT
seq            --------GCACTTTATTTGTAATGAGGGTTATTACTTAATTGGTGAAGAAATTCTATAT
                       ****************************************************

CD46-005/006   TGTGAACTTAAAGGATCAGTAGCAATTTGGAGCGGTAAGCCCCCAATATGTGAAAAGGTT
seq            TGTGAACTTAAAGGATCAGTAGCAATTTGGAGCGGTAAGCCCCCAATATGTGAAAAGGTT
               ************************************************************

CD46-005/006   TTGTGTACACCACCTCCAAAAATAAAAAATGGAAAACACACCTTTAGTGAAGTAGAAGTA
seq            TTGTGTACACCACCTCCAAAAATAAAAAATGGAAAACACACCTTTAGTGAAGTAGAAGTA
               ************************************************************

CD46-005/006   TTTGAGTATCTTGATGCAGTAACTTATAGTTGTGATCCTGCACCTGGACCAGATCCATTT
seq            TTTGAGTATCTTGATGCAGTAACTTATAGTTGTGATCCTGCACCTGGACCAGATCCATTT
               ************************************************************

CD46-005/006   TCACTTATTGGAGAGAGCACGATTTATTGTGGTGACAATTCAGTGTGGAGTCGTGCTGCT
seq            TCACTTATTGGAGAGAGCACGATTTATTGTGGTGACAATTCAGTGTGGAGTCGTGCTGCT
               ************************************************************

CD46-005/006   CCAGAGTGTAAAGTGGTCAAATGTCGATTTCCAGTAGTCGAAAATGGAAAACAGATATCA
seq            CCAGAGTGTAAAGTGGTCAAATGTCGATTTCCAGTAGTCGAAAATGGAAAACAGATATCA
               ************************************************************

CD46-005/006   GGATTTGGAAAAAAATTTTACTACAAAGCAACAGTTATGTTTGAATGCGATAAGGGTTTT
seq            GGATTTGGAAAAAAATTTTACTACAAAGCAACAGTTATGTTTGAATGCGATAAGGGTTTT
               ************************************************************

CD46-005/006   TACCTCGATGGCAGCGACACAATTGTCTGTGACAGTAACAGTACTTGGGATCCCCCAGTT
seq            TACCTCGATGGCAGCGACACAATTGTCTGTGACAGTAACAGTACTTGGGATCCCCCAGTT
               ************************************************************

CD46-005/006   CCAAAGTGTCTTAAAGGTCCTAGGCCTACTTACAAGCCTCCAGTCTCAAATTATCCAGGA
seq            CCAAAGTGTCTTAAAGGTCCTAGGCCTACTTACAAGCCTCCAGTCTCAAATTATCCAGGA
               ************************************************************

CD46-005/006   TATCCTAAACCTGAGGAAGGAATACTTGACAGTTTGGATGTTTGGGTCATTGCTGTGATT
seq            TATCCTAAACCTGAGGAAGGAATACTTGACAGTTTGGATGTTTGGGTCATTGCTGTGATT
               ************************************************************

CD46-005/006   GTTATTGCCATAGTTGTTGGAGTTGCAGTAATTTGTGTTGTCCCGTACAGATATCTTCAA
seq            GTTATTGCCATAGTTGTTGGAGTTGCAGTAATTTGTGTTGTCCCGTACAGATATCTTCAA
               ************************************************************

CD46-005/006   AGGAGGAAGAAGAA-AGGCACATACCTAACTGATGAGACCCACAGAGAAGTAAAATTTAC
seq            AGGAGGAAGAAAAAAAGGCA----------------------------------------
               ***********.** *****
```

Supplementary DNA sequence 1

Cloned RT-PCR CD46 splice variants from piglet 1706 aligned with ENSEMBL reference sequences for CD46 variants 001/007, 002/004 and 005/006, as indicated

```
CD55-001      GGAGAGCACTCTATTTATTGTACTGTGAATAATGATGAAGGAGAGTGGAGTGGCCCACCA
seq           --------------------------------TG-ATGAGGAGAGTGGAGTGGCCCACCA
                                              ** : .**********************

CD55-001      CCTGAATGCAGAGGAAAATCTCTAACTTCCAAGGTCCCACCAACAGTTCAGAAACCTACC
seq           CCTGAATGCAGAGGAAAATCTCTAACTTCCAAGGTCCCACCAACAGTTCAGAAACCTACC
              ************************************************************

CD55-001      ACAGTAAATGTTCCAACTACAGAAGTCTCACCAACTTCTCAGAAAACCACCACAAAAACC
seq           ACAGTAAATGTTCCAACTACAGAAGTCTCACCAACTTCTCAGAAAACCACCACAAAAACC
              ************************************************************

CD55-001      ACCACACCAAATGCTCAAGCAACACGGAGTACACCTGTTTCCAGGACAACCAAGCATTTT
seq           ACCACACCAAATGCTCAAGCAACACGGAGTACACCTGTTTCCAGGACAACCAAGCATTTT
              ************************************************************

CD55-001      CATGAAACAACCCCAAATAAAGGAAGTGGAACCACTTCAGGTACTACCCGTCTTCTATCT
seq           CATGAAACAACCCCAAATAAAGGAAGTGGAACCACTTCAGGTACTACCCGTCTTCTATCT
              ************************************************************

CD55-001      GGGCACACGTGTTTCACGTTGACAGGTTTGCTTGGGACGCTAGTAACCATGGGCTTGCTG
seq           GGGCACACGTGTTTCACGTTGACAGGTTTGCTTGGGACGCTAGTAACCATGGGCTTGCTG
              ************************************************************

CD55-001      ACTTAG
seq           ACTT--
              ****
```

```
CD55-009      GGAGAGCACTCTATTTATTGTACTGTGAATAATGATGAAGGAGAGTGGAGTGGCCCACCA
seq           -----------------------------ATATG-ATGAGGAGAGTGGAGTGGCCCACCA
                                           ::*** : .**********************

CD55-009      CCTGAATGCAGAGGAAAATCTCTAACTTCCAAGGTCCCACCAACAGTTCAGAAACCTACC
seq           CCTGAATGCAGAGGAAAATCTCTAACTTCCAAGGTCCCACCAACAGTTCAGAAACCTACC
              ************************************************************

CD55-009      ACAGTAAATGTTCCAACTACAGAAGTCTCACCAACTTCTCAGAAAACCACCACAAAAACC
seq           ACAGTAAATGTTCCAACTACAGAAGTCTCACCAACTTCTCAGAAAACCACCACAAAAACC
              ************************************************************

CD55-009      ACCACACCAAATGCTCAAGCAACACGGAGTACACCTGTTTCCAGGACAACCAAGCATTTT
seq           ACCACACCAAATGCTCAAGCAACACGGAGTACACCTGTTTCCAGGACAACCAAGCATTTT
              ************************************************************

CD55-009      CATGAAACAACCCCAAATAAAGGAAGTGGAACCACTTCAGGTACTACCCGTCTTCTATCT
seq           CATGAAACAACCCCAAATAAAGGAAGTGGAACCACTTCAGGTACTACCCGTCTTCTATCT
              ************************************************************

CD55-009      GGTTCTCGTCCTGTCACCCAGGCTGGTATGCGGTGGTGTGATCGTAGCTCACTGCAGTCT
seq           GGTTCTCGTCCTGTCACCCAGGCTGGTATGCGGTGGTGTGATCGTAGCTCACTGCAGTCT
              ************************************************************

CD55-009      CGAACTCCTGGGTTCAAGCGATCCTTCCACTTCAGCCTCCCAAGTAGCTGGTACTACAGG
seq           CGAACTCCTGGGTTCAAGCGATCCTTCCACTTCAGCCTCCCAAGTAGCTGGTACTACAGG
              ************************************************************

CD55-009      GCACACGTGTTTCACGTTGACAGGTTTGCTTGGGACGCTAGTAACCATGGGCTTGCTGAC
seq           GCACACGTGTTTCACGTTGACAGGTTTGCTTGGGACGCTAGTAACCATGGGCTTGCTGAC
              ************************************************************

CD55-009      TTAGCCAAAGAAGAGTTAAGAAGAAAATACACACAAGTATACAGACTGTTCCTAGTTTCT
seq           TA----------------------------------------------------------
              *:
```

Supplementary DNA sequence 2

Cloned RT-PCR CD55 splice variants from piglet 1706 aligned with ENSEMBL reference sequences for CD55 variants 001 (gDAF) and 009 (sDAF), as indicated
